# The balance between gyrase and topoisomerase I activities determines levels of supercoiling, nucleoid compaction, and viability in bacteria

**DOI:** 10.3389/fmicb.2022.1094692

**Published:** 2023-01-11

**Authors:** Míriam García-López, Diego Megias, María-José Ferrándiz, Adela G. de la Campa

**Affiliations:** ^1^Unidad de Genética Bacteriana, Centro Nacional de Microbiología, Instituto de Salud Carlos III, Majadahonda, Madrid, Spain; ^2^Unidad de Microscopía Confocal, Instituto de Salud Carlos III, Majadahonda, Madrid, Spain; ^3^Presidencia, Consejo Superior de Investigaciones Científicas, Madrid, Spain

**Keywords:** DNA supercoiling, DNA topoisomerase I, DNA gyrase, regulation of supercoiling, supercoiling homeostasis

## Abstract

Two enzymes are responsible for maintaining supercoiling in the human pathogen *Streptococcus pneumoniae*, gyrase (GyrA_2_GyrB_2_) and topoisomerase I. To attain diverse levels of topoisomerase I (TopoI, encoded by *topA*), two isogenic strains derived from wild-type strain R6 were constructed: P_Zn_*topA*, carrying an ectopic *topA* copy under the control of the ZnSO_4_-regulated P_Zn_ promoter and its derivative Δ*topA*P_Zn_*topA*, which carries a *topA* deletion at its native chromosomal location. We estimated the number of TopoI and GyrA molecules per cell by using Western-blot and CFUs counting, and correlated these values with supercoiling levels. Supercoiling was estimated in two ways. We used classical 2D-agarose gel electrophoresis of plasmid topoisomers to determine supercoiling density (σ) and we measured compaction of nucleoids using for the first time super-resolution confocal microscopy. Notably, we observed a good correlation between both supercoiling calculations. In R6, with σ = −0.057, the average number of GyrA molecules per cell (2,184) was higher than that of TopoI (1,432), being the GyrA:TopoI proportion of 1:0.65. In Δ*topA*P_Zn_*topA*, the number of TopoI molecules depended, as expected, on ZnSO_4_ concentration in the culture media, being the proportions of GyrA:TopoI molecules in 75, 150, and 300 μM ZnSO_4_ of 1:0.43, 1:0.47, and 1:0.63, respectively, which allowed normal supercoiling and growth. However, in the absence of ZnSO_4_, a higher GyrA:TopoI ratio (1:0.09) caused hyper-supercoiling (σ = −0.086) and lethality. Likewise, growth of Δ*topA*P_Zn_*topA* in the absence of ZnSO_4_ was restored when gyrase was inhibited with novobiocin, coincidentally with the resolution of hyper-supercoiling (σ change from −0.080 to −0.068). Given that TopoI is a monomer and two molecules of GyrA are present in the gyrase heterotetramer, the gyrase:TopoI enzymes proportion would be 1:1.30 (wild type R6) or of 1:1.26–0.86 (Δ*topA*P_Zn_*topA* under viable conditions). Higher proportions, such as 1:0.18 observed in Δ*topA*P_Zn_*topA* in the absence of ZnSO_4_ yielded to hyper-supercoiling and lethality. These results support a role of the equilibrium between gyrase and TopoI activities in supercoiling maintenance, nucleoid compaction, and viability. Our results shed new light on the mechanism of action of topoisomerase-targeting antibiotics, paving the way for the use of combination therapies.

## Introduction

*Streptococcus pneumoniae* is a main cause of community-acquired pneumonia, meningitis, bacteremia, and otitis media, causing annually the death of 1 million children worldwide (World Health Organization, 2007). Resistance in this bacterium to beta-lactam and macrolide antibiotics has spread globally (Jacobs et al., 2003). However, low levels of resistance have been detected for fluoroquinolones (Domenech et al., 2014), which are directed to type II DNA topoisomerases. These drugs are nowadays recommended for treatment of pneumococcal infections (Mandell et al., 2007).

DNA topoisomerases are essential for supercoiling (Sc) maintenance, which in turn is essential for cell viability. *S. pneumoniae* possesses three topoisomerases, two of type II (topoisomerase IV and gyrase) and a single type I enzyme, topoisomerase I (TopoI). These three enzymes solve topological problems associated with dynamic DNA remodeling (Ferrándiz et al., 2010; de la Campa et al., 2017). Bacteria maintain Sc homeostasis by regulating transcription of their topoisomerase genes. First evidences of Sc homeostasis were reported in *Escherichia coli*. DNA relaxation in this bacterium decreases transcription of the TopoI coding gene (Tse-Dinh, 1985) and increases those coding for gyrase (Menzel and Gellert, 1983, 1987a,b) to get an equilibrium in Sc. Sc also regulates transcription of topoisomerases in *S. pneumoniae* as part of a general gene regulatory mechanism. The pneumococcal genome is organized into Sc domains, in which genes show a coordinated transcription, independently of their transcription orientation (Ferrándiz et al., 2010, 2016). Genes of the same Sc domain have also similar functions (Martín-Galiano et al., 2017). When Sc density decreases, i.e., DNA is more relaxed, a response showing transcriptional domains is induced. Domains containing genes with decreased transcription are nominated DOWN (downregulated), and those containing genes with increased transcription are nominated UP (upregulated). Genes encoding topoisomerases are themselves located in Sc domains: *topA* in a DOWN domain (Ferrándiz et al., 2010), and *gyrB* in an UP domain (Ferrándiz et al., 2014). In fact, TopoI plays a fundamental role in the regulation of transcription by Sc. Transcription levels of *topA* in homeostasis correlates with the induced variation in the density of Sc (Ferrándiz et al., 2016).

Sc regulates transcription, and transcription in turn is a major contributor to the level of Sc. The twin supercoiled-domain model proposes that domains of negative and positive Sc are transiently generated behind and ahead of the moving RNA polymerase, respectively (Liu and Wang, 1987). A physical interaction of TopoI and RNAP has been detected *in vitro* both in *E. coli* (Cheng et al., 2003) and in *S. pneumoniae* (Ferrándiz et al., 2021). ChIP-Seq experiments have shown co-localization of RNAP, TopoI, and gyrase on the active transcriptional units of *Mycobacterium tuberculosis* (Ahmed et al., 2017). In addition, we have recently shown a genome-wide proximity between TopoI and RNA polymerase using ChIP-Seq, supporting the interplay between transcription and supercoiling, and the role of TopoI in the formation/stability of the RNAP-DNA complex at the promoter during transcript elongation (Ferrándiz et al., 2021).

The essentiality of TopoI for viability has been extensively studied in *E. coli* and closely related bacteria, such as *Salmonella enterica* and *Shigella flexneri* (Richardson et al., 1984; Ni Bhriain and Dorman, 1993; Stockum et al., 2012). TopoI deficiency has been associated with the accumulation of negative Sc and increased prevalence of R-loops (DNA–RNA hybrids) that serve as sites for aberrant chromosomal replication (Brochu et al., 2020). The toxicity induced is alleviated by compensatory mechanisms that include mutations in gyrase genes (DiNardo et al., 1982), or overexpression of Topo III (Broccoli et al., 2000), the paralog of Topo I, or Topo IV (McNairn et al., 1995). In turn, the increased formation of R-loops that leads to RNA backtracking at the site of conflict is relieved by the overexpression of RNase HI (Drolet et al., 1995). TopoI is essential in different species of mycobacteria irrespective of whether they possess a sole enzyme or have additional DNA relaxation enzymes (Ahmed et al., 2015). In *S. pneumoniae*, which only has one type IA topoisomerase, as *M. tuberculosis*, the lack of this enzyme it is supposed to be deleterious.

In our previous RNA-Seq and CHIP-Seq investigations, we have established that the transcriptional regulation of *topA* is essential for Sc control and transcription. In this study, we make genetic constructions that allow Sc-independent regulation of TopoI expression and study the effects of this deregulation in cell viability. We correlate the level of TopoI with cell growth and Sc density. Our results showed a fundamental role for TopoI in Sc maintenance and cell viability. Knowledge of the mechanisms of Sc maintenance is essential to establish an adequate antibiotic therapy, which might include drug combinations.

## Materials and methods

### Microbiological methods and genetic constructions

*S. pneumoniae* was grown at 37°C in a casein hydrolysate-based liquid medium (AGCH) in which ZnSO_4_ was depleted and adjusted according to the experimental needs. This medium also contained 0.2% yeast extract and 0.3% sucrose (Lacks et al., 1986). Transformation was performed as previously reported (Lacks et al., 1986). Selection of transformants was made in 1 μg/ml tetracycline, 250 μg/ml kanamycin, or 2.5 μg/ml chloramphenicol when strains were transformed with plasmid pLS1, the kanamycin resistance cassette (*kan*), or the chloramphenicol resistance cassette (*cat*), respectively. To induce DNA relaxation, 0.25 μg/ml (0.25 × MIC) of novobiocin (Nov) was added to the culture. Growth was monitored by measuring OD_620nm_ either in an UV–visible spectrophotometer (Evolution 201, Thermo Scientific) or in a microplate reader (Infinite F200, Tecan). Both measures correlate linearly by means of the equation *y* = 0.2163 *x* + 0.1151 (*y* = microplate reader measure, *x* = spectrophotometer), with an *R*^2^ of 0.98 (Ferrándiz et al., 2018).

**Figure 1:**
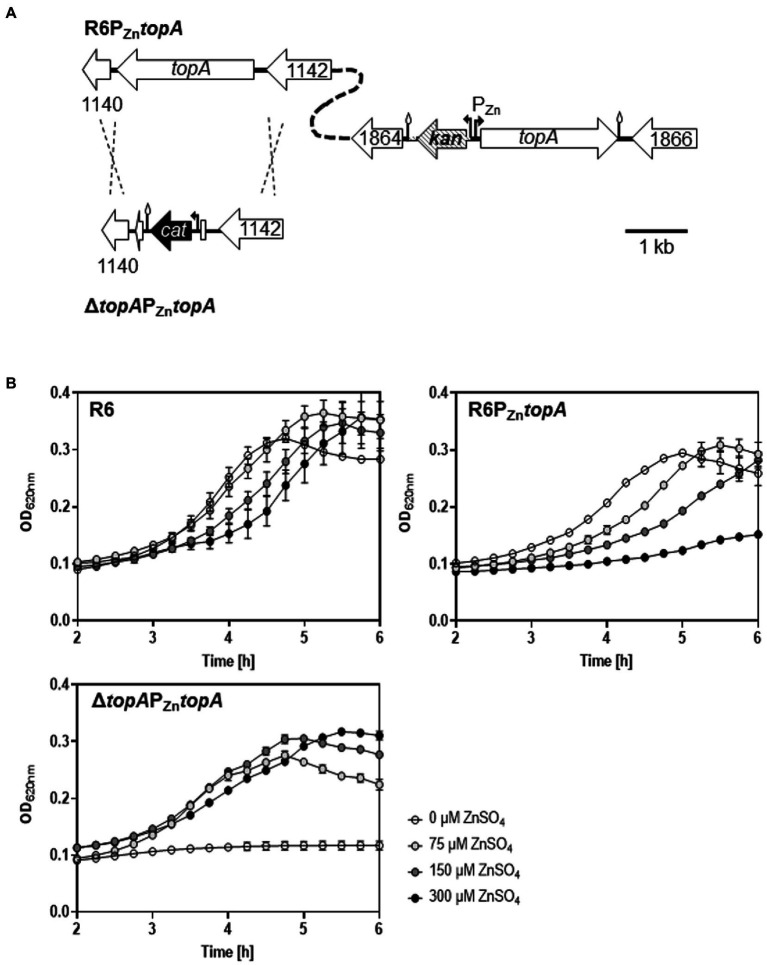
Levels of TopoI affect growth. **(A)** Representation of the genetic structure of the R6-derivative strains with respect the location of the *topA* gene. **(B)** Growth of the indicated strains monitored in a TECAN Infinite 200 PRO reader. Strains were grown to OD_620nm_ = 0.4 in medium containing 75 μM ZnSO_4_ and diluted 100-fold in medium containing the indicated amounts of ZnSO_4_.

Strain P_Zn_*topA* (Figure 1A) containing *topA* (spr1141) under the control of the P_Zn_ promoter at the spr1865 locus, which is not esssential for growth (Martín-Galiano et al., 2014), was constructed as follows. Three DNA fragments were obtained by PCR. All primers are detailed in Table 1. The first PCR product (2,157 pb) included *topA* flanked by restriction sites present in primers TOPAUPSAC (SacI) and TOPADOWNSAL(SalI), which were used to amplify *topA* from R6 chromosome. The second product (671 pb) contained a consensus transcriptional terminator followed by the C-terminus of spr1866 amplified from the chromosome of R6P_Zn_*hlp*Δ*hlp* (Ferrándiz et al., 2018) with primers SPR1866R and TERSAL (SalI). The third PCR product (1,704 pb) contained P_Zn_, *kan*, the N-terminus of spr1865, spr1864, and spr1863. It was also amplified from R6P_Zn_*hlp*Δ*hlp* with primers PZNRSAC (SacI) and SPR1863F. These three PCR products were digested and ligated. The ligation product was used as a template to obtain a 4,532-bp PCR product with the oligonucleotide pair SPR1866R/SPR1863F, which was used to transform R6 competent cells. Transformants were selected by plating in AGCH-agar medium without ZnSO_4_ (to avoid overexpression of *topA*) containing 250 μg/ml kanamycin. To confirm the insertion in spr1865, amplification from the chromosome was performed with primers SPR1866R2 and SPR1863F2 flanking the inserted DNA. Primers TOPAUP2, TOPARTF, TOPARTR, and TOPADOWN were used to sequence P_Zn_*topA* insertion.

**Table 1:** Oligonucleotides used in this study.

Primer name	Sequence (5′ → 3′)^a^	Nucleotide positions^b^
CAT191	GTGATGGTTATCATGCAGG	575–593 of *cat*
CATDOWNHIN	cgcgcaagcttGATATGGATCTGGAGCTGTAA	734–753 of *cat*
CATMED	CCTAACTCTCCGTCGCTATTG	Complementary to 213–232 of *cat*
ECOCATUP	gcgcggaattcGCACCCATTAGTTCAACAAACG	−165 to −185 of *cat*
GYRARTF	TGATAAACGCCGTACAGAGTT	1,431–1,451 of *gyrA*
GYRARTR	CCACGACCCCCACGTTTTTGAGC	Complementary to 1,567–1,589 of *gyrA*
PZNRSAC	cgcgcgagctcTCTTATTTCTCATTC	-
RPOB428	CGGTTGGTGAATTGCTTGCCAACCA	1,283–1,307 of *rpoB*
RPOB474R	ACTGCAGCTGTTACAGGACGG	Complementary to 1,404–1,424 of *rpoB*
SPR1863F	CCACTCCCAACCGGACCAGCA	389–410 of *spr1863*
SPR1863F2	GATTAATCTCTGGTAGCAGACT	20–41 of *spr1863*
SPR1866R	GTAGACCTAGACGATAACCGC	Complementary to 591–612 of *spr1866*
SPR1866R2	TGGATTTTCGGTCACTTGATTG	Complementary to 328–349 of *spr1866*
TERSAL	cgcgcgtcgacTATAAGAAAAAATGA	-
TOPADOWN	TTTAATCTTTTCTTCCTCGTAG	Complementary to 2,063–2,085 of *spr1141*
TOPADOWNSAL	cgcgcgtcgacTTATTTAATCTTTTCTTCCTC	Complementary to 2,067–2,088 of *spr1141*
TOPAKOF1	TCTGGGAGTGGGGCTCTCTCT	186–206 of *spr1142*
TOPAKOF2	CGTCAGCTCAGCTTTGCCTTG	175–176 of *spr1143*
TOPAKOF3	gcgcgaagcttCCTGTTGGTCGTGACTGTCC	1,972–1,992 of *spr1141*
TOPAKOR2	CGCCAGACACACCAGCACGAG	Complementary to 452–473 of *spr1139*
TOPAKOR3	CAGCAGTGATGGACACAGTCA	Complementary to 119–140 of *spr1138*
TOPAKORI	cgcgcgaattcGGCCTTAGCAGGCGACTCCACG	Complementary to 54–76 of *spr1141*
TOPARTF	TCACCAAGGATGCAGTCAAAAATG	371–394 of *topA*
TOPARTR	GGCGAAATCGAATACCCTACCA	Complementary to 467–488 of *topA*
TOPAUP2	GTGGCTACGGCAACAAAAAAGAA	1–23 of *spr1141*
TOPAUPSAC	cgcgcgagctcGTGGCTACGGCAACAAAAAAG	1–21 of *spr1141*

^a^Lower case indicates bases added to the annealing sequence. Underlined sequences correspond to restriction targets. ^b^Nucleotide numbers refer to the genes of the *Streptococcus pneumoniae* R6 sequence. The first nucleotide of the gene is considered nt 1.

To construct strain Δ*topA*P_Zn_*topA* (Figure 1A), *topA* was disrupted in the chromosome of P_Zn_*topA* by homologous recombination as follows. Three DNA fragments were obtained by PCR amplification. Two fragments upstream and downstream *topA* of 1,103 and 1,149 bp were amplified with primers TOPAKOF1 and TOPAKORI (EcoRI) and TOPAKOF3 and TOPAKOR2 (HindIII), respectively, using R6 DNA as template. A third DNA fragment of 924 bp containing the *cat* cassette was amplified from plasmid pJS3 with primers ECOCATUP (EcoRI) and CATDOWNHIN (HindIII). The three fragments were digested and ligated, and the ligation product was used as a template to obtain a 3,176 bp PCR product (oligonucleotide pair TOPAKOF1/TOPAKOR2), which was used to transform P_Zn_*topA*. Transformants were selected by plating in AGCH-agar medium supplemented with 150 μM ZnSO_4_ (to allow *topA* expression) and 2.5 μg/ml chloramphenicol. To confirm the disruption, amplification from the chromosome was performed with primers TOPAKOF2 and TOPAKOR3 flanking the replaced DNA. To sequence the construct, primers TOPAUP2, TOPARTF, TOPARTR, TOPADOWN, CATMED, and CAT191 were used.

### qRT-PCR

Total RNA was extracted from 10 ml of culture (OD_620nm_ = 0.4) with RNeasy kit (Qiagen), following the manufacturer’s instructions, with the exception that RNA was treated 3-fold with DNase I. cDNAs were synthesized from 5 μg of RNA with SuperScript™ IV Reverse Transcriptase (Thermo-Fisher) for 10 min at 55°C. A total of 2 μl of diluted cDNA (2,000-fold for 16S rDNA and one 20-fold for the rest of amplicons) were used as template in a subsequent qRT-PCR (CFX Opus 96, Bio-Rad) using 10 μl of SsoAdvanced Universal SYBR Green Supermix (Bio-Rad). Amplification was achieved as previously described (Ferrándiz et al., 2010). Primer pairs used (GYRARTF/GYRARTR, TOPARTF/TOPARTR, and 16SDNAF3/16SDNAR3) are indicated in Table 1. Three independent assays were performed for quantification. Analysis of qRT-PCR data was performed using the 2^−ΔΔCq^ method (Livak and Schmittgen, 2001) using an internal fragment of 16S rDNA gene as an internal control and 0 ZnSO_4_ condition expression levels as the calibrator.

### Western-blot

Whole cell lysates (~ 5 × 10^5^ cells) were obtained by centrifugation of a 10-ml culture (OD_620nm_ = 0.4), resuspended in 400 μl of phosphate buffered saline and sonicated for 20 min (30 s ON/30 s OFF) with a Bioruptor® Pico sonication device (Diagenode). Lysates (~ 2 × 10^4^ cells) were separated on Any kD™ Criterion™ TGX Stain-Free™ Protein Gels (Bio-Rad). They were transferred to 0.2 μm PVDF membranes with a Trans-Blot Turbo Transfer System (Bio-Rad) at 25 V, 1 A for 30 min. Membranes were blocked with 5% milk in Tris-buffered saline for 2 h and incubated with anti-TopoI (diluted 1:500), anti-GyrA (diluted 1:2,000; Ferrándiz et al., 2016), and anti-RpoB (Ferrándiz et al., 2021; diluted 1:500). Anti-rabbit IgG-Peroxidase antibody (Sigma-Aldrich) was used as the secondary antibody. SuperSignal West Pico chemiluminescent substrate (Thermo-Fisher) was used to develop the membranes. Signal was detected with a ChemiDocTM MP system (Bio-Rad). Images were analyzed using Image Lab™ software (Bio-Rad). The number of molecules of TopoI and GyrA per cell were estimated by Western blot and by counting CFUs (colony-forming units) at each growth condition. Purified GyrA (2.5 –20 ng) and TopoI (1.5 –24 ng) proteins were used to standarize the correlation between protein amount and immunostaining intensity, which was used to estimate the amount of these proteins in cell lysates. RpoB was used as an internal loading control. For that, an average of the RpoB signal of all wells was calculated, and the deviation of the RpoB signal on each well from this value was assessed. These values were used to adjust TopoI and GyrA signals. CFUs were determined by plating cell extracts on AGCH with 0.2% yeast extract and 0.3% sucrose agar plates. Molecular masses of GyrA and TopoI are 92.04 and 79.38 kDa, respectively. Determinations were performed in triplicate.

### Analysis of the topology of covalently closed circles

Plasmid DNA topoisomers were analyzed in neutral/neutral two-dimensional agarose gels. The first dimension was run at 1.5 V/cm in a 0.4% agarose (Seakem; FMC Bioproducts) gel in 1 × Tris-borate-EDTA (TBE) buffer for 20 h at room temperature. The second dimension was run at 7.5 V/cm in 1% agarose gel in 1 × TBE buffer for 7–9 h at 4°C. Chloroquine (Sigma) was added to the TBE buffer in both, the agarose and the running buffer. Chloroquine is a DNA intercalating agent that removes negative Sc in bacterial plasmids. Increasing concentrations of chloroquine progressively eliminate negative Sc until the plasmid is relaxed and can then introduce net positive Sc. In this way, the use of adequate concentrations of chloroquine during each dimension in the 2D analysis allows the efficient resolution of the different topoisomers (Schvartzman et al., 2013). Gels were stained with 0.5 μg/ml ethidium bromide for 1 h at room temperature. When this staining was not enough for topoisomers visualization, after electrophoresis, gels were subjected to Southern hybridization. A 240-bp PCR fragment amplified from pLS1 obtained as described (Ferrándiz et al., 2010) was used to probe on two-dimensional agarose gels transferred to nylon membrane (Inmobylon NY^+^, Millipore). Streptavidin-HRP (SouthernBiotech) was used to detect the DNA and signal was developed with the SuperSignal West Pico chemiluminescent substrate (Thermo-Fisher). Images were captured in a ChemiDoc Imaging System (Bio-Rad) and analyzed with the Image Lab software (BioRad).

### Nucleoid staining and confocal microscopy

Cultures of strains P_Zn_*topA* and Δ*topA*P_Zn_*topA* were grown at mid-log growth phase in media with different amounts of ZnSO_4_. Samples (from 5 × 10^7^ to 2 × 10^8^ cells) were collected, washed in 10 mM phosphate buffer (pH 7.2), and fixed in 2% of paraformaldehyde for 48 h. Fixed cells were washed, suspended in buffered salt solution (137 mM NaCl, 5.4 mM KCl, 10 mM Tris–HCl, and pH 7.6) and incubated with 5 μM Sytox™ Orange Nucleic Acid Stain (Invitrogen) for 5 min at room temperature. ProLong™ Gold Antifade Mountant (Invitrogen) was added to fixed cells, and the mixture was transferred onto poly-L-lysine coated glass slides (Sigma-Aldrich). Slides were observed using a confocal microscope STELLARIS 8–FALCON/STED (Leica Microsystems) with a HC PL APO 100×/1.40 NA × OIL immersion objective. Super resolution images were acquired by Stimulated Emission Depletion (STED) microscopy using 660_nm_ depletion laser. Image J software was used for image analysis. Average Sytox fluorescence intensity was measured from the nucleoids, defined as the region of each cell with intensity values between 20 and 225. Raw integrated density measurements divided by area (Mean Gray Values) were obtained for 1,307–4,562 nucleoids. GraphPad Prism 9.1 was used to represent the average intensities vs. Sc density (σ) and to perform a simple linear regression.

## Results

### TopoI is essential for growth and its levels govern cell viability

**Figure 2:**
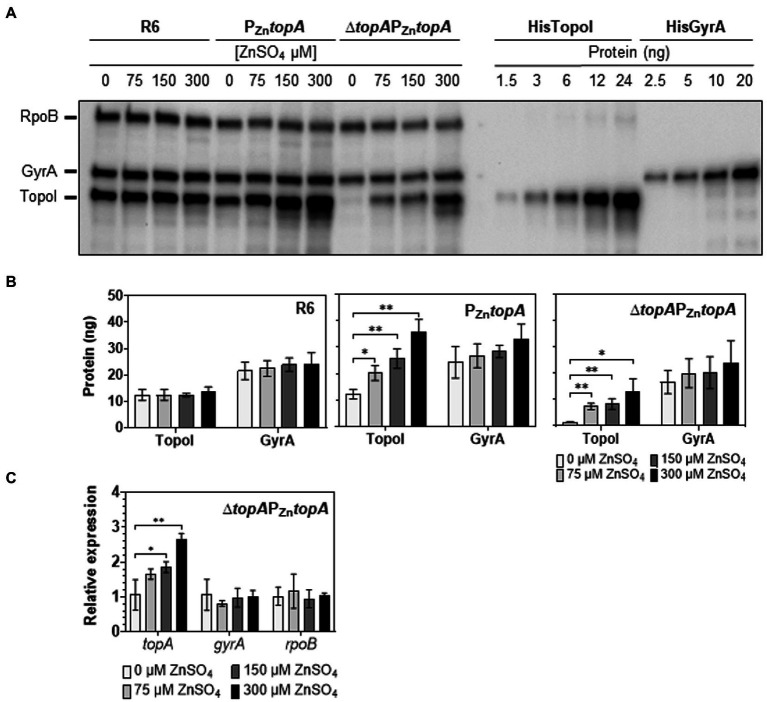
Levels of TopoI in strains carrying P_Zn_*topA* fusions correlate with the concentration of ZnSO_4_ in the growth media. **(A)** A typical Western-blot of TopoI and GyrA is shown. Cells were grown overnight to OD_620nm_ = 0.4 (as measured in the spectrophotometer) in medium containing 75 μM ZnSO_4_. They were diluted 100-fold in media containing the indicated ZnSO_4_ concentrations and regrown to OD_620nm_ = 0.4, except for Δ*topA*P_Zn_*topA* grown without ZnSO_4_ and P_Zn_*topA* grown with 300 μM ZnSO_4_, which samples were taken after 240 min. Samples containing 0.12 units of OD_620nm_ (about 20 μg) were separated by SDS-PAGE and blotted. The membrane was incubated with polyclonal anti-GyrA, anti-TopoI, and anti-RpoB antibodies. The indicated amounts of purified TopoI and GyrA proteins were also analyzed (last nine lines) to use as reference to quantify the amount of these proteins in the crude extracts. **(B)** Quantification of Western-blot experiments. The results (mean ± SD) of three independent replicates are presented. **(C)** Relative expression of *topA* and *gyrA* in Δ*topA*P_Zn_*topA* strain measured by qRT-PCR. Results are the mean ± SD of three independent replicates. Statistical significance two-tailed Student′s *t*-test, ^*^p ≤ 0.05; ^**^p ≤ 0.01.

To ascertain the role of TopoI levels in cell viability, a strain derived from R6 carrying a deletion of *topA* at its native chromosomal location and an ectopic *topA* copy under the control of the P_Zn_ promoter was constructed as described in the section “Materials and methods” (Figure 1A). This strain, named Δ*topA*P_Zn_*topA*, depended on ZnSO_4_ to grow (Figure 1B). It did not grow in the absence of ZnSO_4_, but growth was restored when ≥75 μM ZnSO_4_ was added. On the other hand, strain P_Zn_*topA*, which contains *topA* at its native location and an additional ectopic *topA* copy under the control of P_Zn_, grew normally either in the absence or in the presence of 75 μM of ZnSO_4_, while higher ZnSO_4_ concentrations (150 or 300 μM) compromised its growth. Accordingly, TopoI levels correlated with the concentration of ZnSO_4_ in the growth medium (Figure 2).

The number of TopoI molecules per cell at each condition was estimated by using Western-blot to determine the amount of protein and by counting CFUs. Results are showed in Table 2. In strain Δ*topA*P_Zn_*topA*, a residual amount of TopoI (1.5 ng) was detected in the absence of ZnSO_4_, while the number of molecules increased to 665, 848, and 1,247 in the presence of 75, 150, and 300 μM ZnSO_4_, respectively (Table 2). At 300 μM ZnSO_4_, the number of TopoI molecules was similar to the values estimated for R6 at any ZnSO_4_ concentration. The highest number of TopoI molecules was detected in P_Zn_*topA*; being of 2,879 with 150 μM and 3,352 with 300 μM of ZnSO_4_. This high number of TopoI molecules exerted a negative effect on the growth of this strain (Figure 1B) compared with its growth at either no ZnSO_4_ (1,726) or 75 μM of ZnSO_4_ (2,250).

**Table 2:** Estimation of protein amounts and number of molecules for GyrA and TopoI.

Strain	ZnSO4 (μM)	Protein amount (ng)		Number of molecules	
		GyrA	TopoI	GyrA	TopoI
R6	0	21.6 ± 3.3	12.4 ± 2.1	2,046 ± 315	1,364 ± 225
	75	22.5 ± 3.0	13.8 ± 1.7	2,133 ± 285	1,523 ± 187
	150	23.9 ± 2.5	12.4 ± 0.7	2,266 ± 241	1,359 ± 71
	300	24.1 ± 4.3	13.5 ± 2.0	2,290 ± 406	1,481 ± 219
P_Zn_*topA*	0	24.3 ± 6.0	12.2 ± 1.8	2,971 ± 734	1,726 ± 255
	75	26.7 ± 4.5	20.2 ± 2.8	2,567 ± 435	2,250 ± 315
	150	28.4 ± 2.4	25.7 ± 3.7	2,739 ± 227	2,879 ± 411
	300	33.1 ± 5.7	35.9 ± 4.6	2,664 ± 459	3,352 ± 431
Δ*topA*P_Zn_*topA*	0	16.7 ± 4.4	1.5 ± 0.2	Nd	Nd
	75	20.1 ± 5.4	6.4 ± 1.2	1,555 ± 422	665 ± 109
	150	20.3 ± 6.1	8.3 ± 2.1	1,783 ± 532	848 ± 210
	300	24.0 ± 8.4	13.1 ± 4.9	1,961 ± 686	1,247 ± 460

No ZnSO_4_-dependent variation in the number of gyrase molecules was detected in strains R6, P_Zn_*topA* or Δ*topA*P_Zn_*topA* (Table 2). The ratio of GyrA:TopoI molecules in R6 was of 1:0.66, 1:0.71, 1:0.60, and 1:0.65 when grown in 0, 75, 150, and 300 μM ZnSO_4_, respectively. In P_Zn_*topA*, the GyrA:TopoI ratio experienced a progressive reduction as ZnSO_4_ concentration increased, until a value of 1:1.26 at 300 μM, while half the proportion was observed in R6 strain at the same ZnSO_4_ concentration. This is in agreement with the progressive reduction of cell growth of R6P_Zn_*topA* at increasing ZnSO_4_ concentration compared to R6 strain (Figure 1B). GyrA:TopoI ratio was higher in Δ*topA*P_Zn_*topA* than in R6 when ZnSO_4_ concentrations of 75 or 150 μM ZnSO_4_ were used. The highest proportion (1:0.09, calculated with protein amount) was observed in this strain grown without ZnSO_4_.

Consistent with the variation of TopoI levels in Δ*topA*P_Zn_*topA*, *topA* expression, measured by qRT-PCR, progressively increased as higher ZnSO_4_ concentrations in the medium were used (Figure 2C). The expression level of *topA* in medium with 300 μM of ZnSO_4_ was 1.6-fold higher than that in the presence of 75 μM ZnSO_4_, this is, 2.7 vs. 1.7. This increase was consistent with the 1.8-fold increase observed by Western-blot (Figure 2A). The expression levels of *gyrA* and *rpoB* were independent of ZnSO_4_ (Figure 2C).

These results show that the constructed strains allow to control the level of TopoI inside the cell by ZnSO_4_ addition in a Sc-independent manner, from very low to wild type levels, in Δ*topA*P_Zn_*topA*, and to near double the wild type levels in P_Zn_*topA*. Both strains were further used to assess the effect of TopoI levels in Sc and cell growth.

### Sc levels correlate with the levels of TopoI

**Figure 3:**
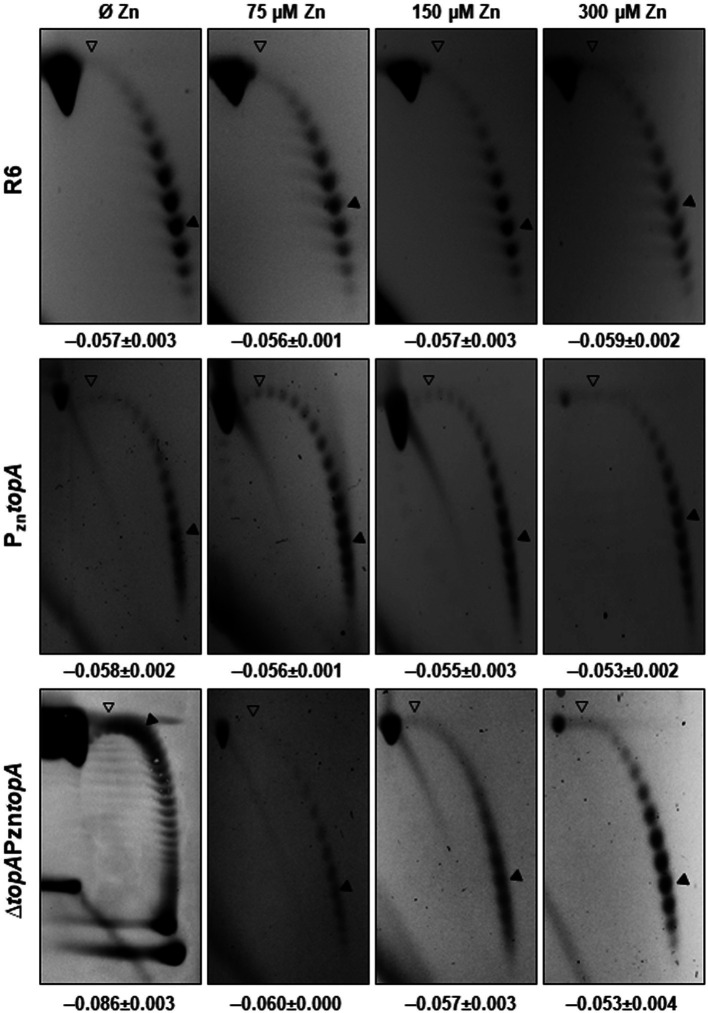
pLS1 topoisomers distribution in the diverse ZnSO_4_ concentrations in strains R6, P_Zn_*topA* and Δ*topA*P_Zn_*topA*. Cultures were grown as in Figure 2. For sample Δ*topA*P_Zn_*topA* 0 ZnSO_4_, 2D-agarose gels were run in the presence of 5 and 15 μg/ml chloroquine in the first and second dimensions, respectively. For the rest of the samples, 1 and 2 μg/ml chloroquine were used. An empty arrowhead indicates the topoisomer that migrated with Δ*Lk* = 0 in the second dimension and has a Δ*Wr* = −14 or −31 during the first dimension (the number of positive supercoils introduced by 2 μg/ml or 15 μg/ml chloroquine), respectively. A black arrowhead indicates the most abundant topoisomer. Mean of three independent experiments ± SD are shown.

We investigated the levels of Sc in the aforementioned strains, Δ*topA*P_Zn_*topA* and P_Zn_*topA*. Measurement of Sc density (σ) of the plasmid pLS1 present in the cells was carried out as described in the section “Materials and methods.” In the presence of residual amounts of TopoI (0 ZnSO_4_), a Sc density (σ) of −0.086 was observed in strain Δ*topA*P_Zn_*topA* (Figure 3). When the number of TopoI molecules increased to 665 (75 μM ZnSO_4_), 848 (150 μM ZnSO_4_), or 1,247 (300 μM ZnSO_4_), the density of negative Sc decreased to values of −0.060, −0.057, and −0.053, respectively, similar to σ values observed in R6 and R6P_Zn_*topA* (Figure 3). Therefore, the variation of TopoI levels at the different ZnSO_4_ concentrations tested was consistent with the Sc level.

### The increased Sc density of Δ*topA*P_Zn_*topA* allowed a higher level of resistance to novobiocin

**Figure 4:**
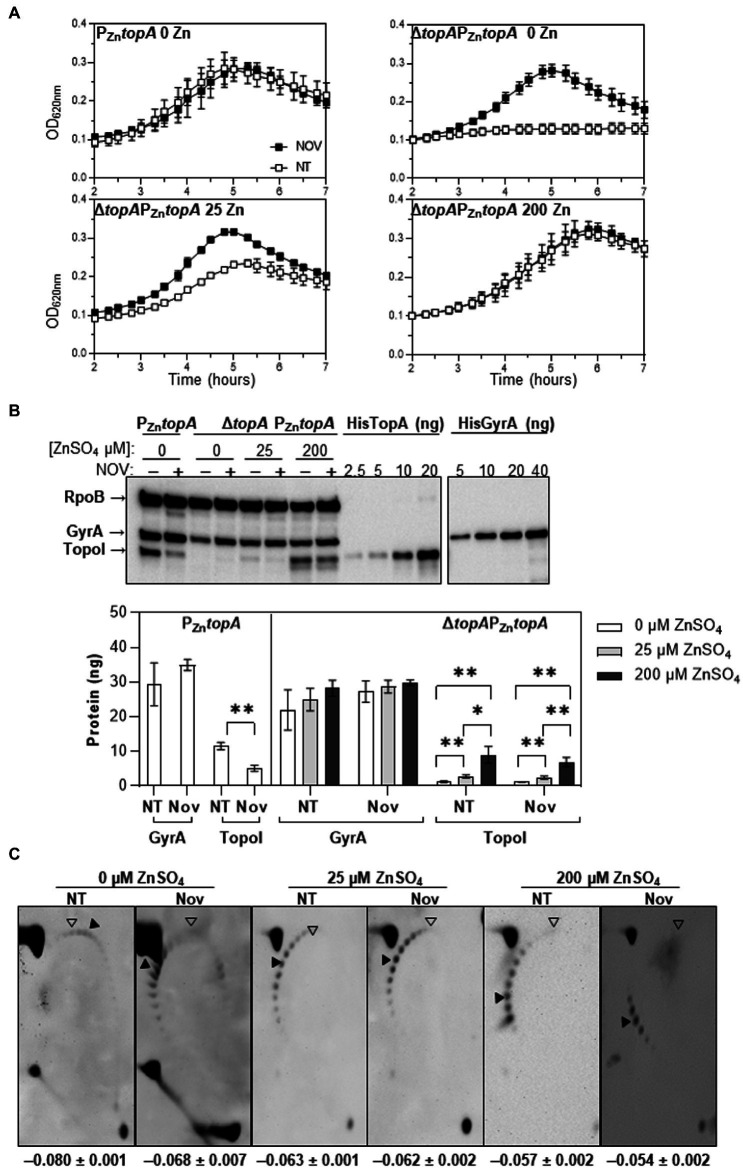
Effect of the treatment of Δ*topA*P_Zn_*topA* with Nov on growth and Sc. Strains P_Zn_*topA* and Δ*topA*P_Zn_*topA* were grown either in media without or with 150 μM ZnSO_4_, respectively, until OD_620nm_ = 0.4 (spectrophotometer measurement). Cells were washed and diluted 100-fold in media containing diverse ZnSO_4_ concentrations. Cultures were either not treated (NT) or treated with Nov at 0.25 × MIC. Samples were taken at OD_620nm_ = 0.15–0.4, except for Δ*topA*P_Zn_*topA* grown without ZnSO_4_, in which samples were taken after 240 min. **(A)** Growth curves of indicated strains monitored in a TECAN Infinite 200 PRO reader. **(B)** Western-blot analysis of GyrA and TopoI proteins. Experiments were performed as described in Figure 2 legend. **(C)** 2D-agarose gel electrophoresis of plasmid DNAs. Chloroquine concentrations used in the first and the second dimension were 5 and 15 μg/ml, respectively. Sc density (σ) values are shown (averages ± SD). An empty arrowhead indicates the topoisomer that migrated with Δ*Lk* = 0 in the second dimension and that had a Δ*Wr* of −31 (the number of positive supercoils introduced by 15 μg/ml chloroquine). A black arrowhead points to the more abundant topoisomer. Results are the mean ± SD of three independent replicates. Statistical significance two-tailed Student′s *t*-test, ^*^p ≤ 0.05; ^**^p ≤ 0.01.

The growth of strain Δ*topA*P_Zn_*topA* in the presence of novobiocin (Nov), which targets gyrase, was studied. To modulate the levels on TopoI expression, this strain was grown in media with either 0, 25, or 200 μM of ZnSO_4_ (Figure 4A). Its parental strain (P_Zn_*topA*) grown in medium without ZnSO_4_ was used as a control. As already seen in this study, Δ*topA*P_Zn_*topA* was unable to grow in the absence of ZnSO_4_. Notably, treatment with a subinhibitory Nov concentration (0.25 × MIC), restored growth in the absence of ZnSO_4_, with a duplication time of 91 ± 6 min, similar to that of P_Zn_*topA* treated with Nov (82 ± 3 min; Figure 4A). Although Δ*topA*P_Zn_*topA* in media with 25 μM of ZnSO_4_ grew slower (96 ± 3 min) than P_Zn_*topA* without ZnSO_4_ (75 ± 9 min), addition of Nov reduced the duplication time to 77 ± 5 min, close to that of untreated P_Zn_*topA*.

Growth of Δ*topA*P_Zn_*topA* in medium with the three concentrations of ZnSO_4_ studied above, rendered three levels of TopoI expression as shown by Western-blot (Figure 4B): nearly no protein without ZnSO_4_, a low amount of TopoI with 25 μM ZnSO_4_, and an amount equivalent to that of the control strain with 200 μM ZnSO_4_. Without Nov treatment (NT), in the absence of ZnSO_4_, a faint band of TopoI was detected (1.2 ± 0.3 ng) in strain Δ*topA*P_Zn_*topA*, which represented about 10% the amount of TopoI observed in P_Zn_*topA* control strain (11.6 ± 1.1 ng). In contrast, in the presence of 25 μM ZnSO_4_, a low but higher amount of TopoI (2.8 ± 0.4 ng) was detected, which represented 24% the TopoI present in the control strain. Under 200 μM of ZnSO_4_, the amount of TopoI (9.0 ± 2.4 ng) was close to that of the control strain (76%). Treatment of the control strain with Nov (0.25 × MIC) decreased 2.2-fold the amount of TopoI due to DNA relaxation. As expected, no change in the amount of TopoI was induced by Nov in Δ*topA*P_Zn_*topA* under any growth conditions since *topA* expression was under the control of P_Zn_, whose activity does not dependent on the Sc level.

Measurement of Sc density of plasmid pLS1 present in Δ*topA*P_Zn_*topA* in the absence of TopoI expression was −0.080 and for Δ*topA*PZn*topA* grown in 200 μM of ZnSO_4_, was of −0.057. When Δ*topA*PZn*topA* was grown in the absence of ZnSO_4_, but the presence of Nov, DNA relaxed by 15.0% (σ values from −0.080 to −0.068), consistent with the growth restoration observed (Figure 4C). Therefore, the release of DNA Sc *via* addition of either ZnSO_4_ or Nov allowed restoration of growth (Figure 4C).

### Correlation between Sc density estimated by 2D-gel electrophoresis of plasmid topoisomers and nucleoid compaction determined by confocal microscopy

**Figure 5:**
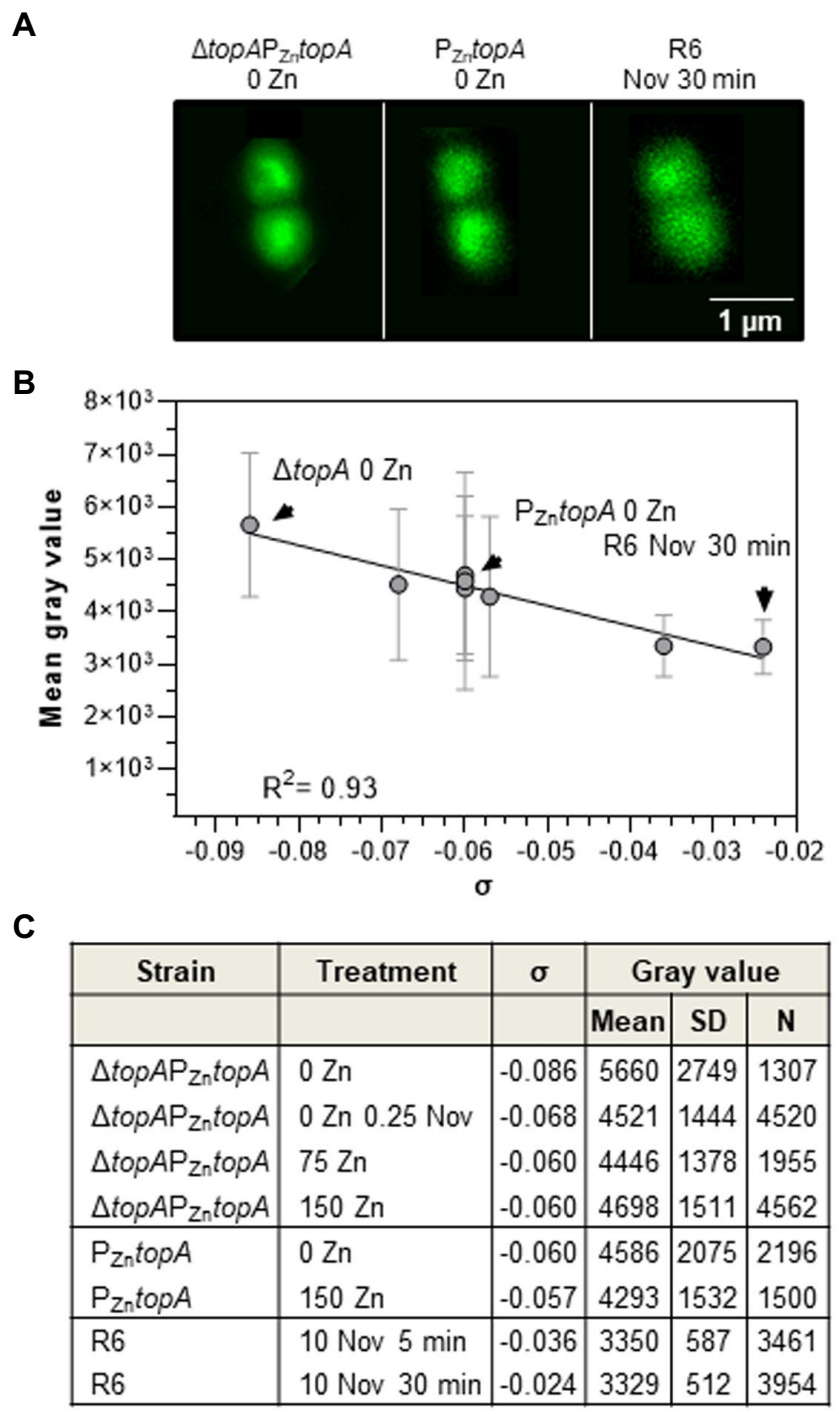
Nucleoid compaction measurement by using super-resolution confocal microscopy. Cells grown as in Figure 2 were fixed and stained with Sytox orange as described in the section “Materials and methods.” Additionally, a culture of strain R6 was grown until OD_620nm_ = 0.4 and treated with 10 × MIC of Nov for 5 or 30 min. **(A)** Stimulated emission depletion (STED) microscopy images of cells indicated. **(B)** Correlation between values obtained in confocal microscopy vs. σ values estimated in 2D-agarose gels of strains and treatments indicated in **(C)**. Mean values ±SD are represented. Points corresponding to images in **(A)** are indicated with an arrow. **(C)** Table showing data represented in **(B)**, indicating mean gray value, SD, and number of cells studied (N).

**Figure 6:**
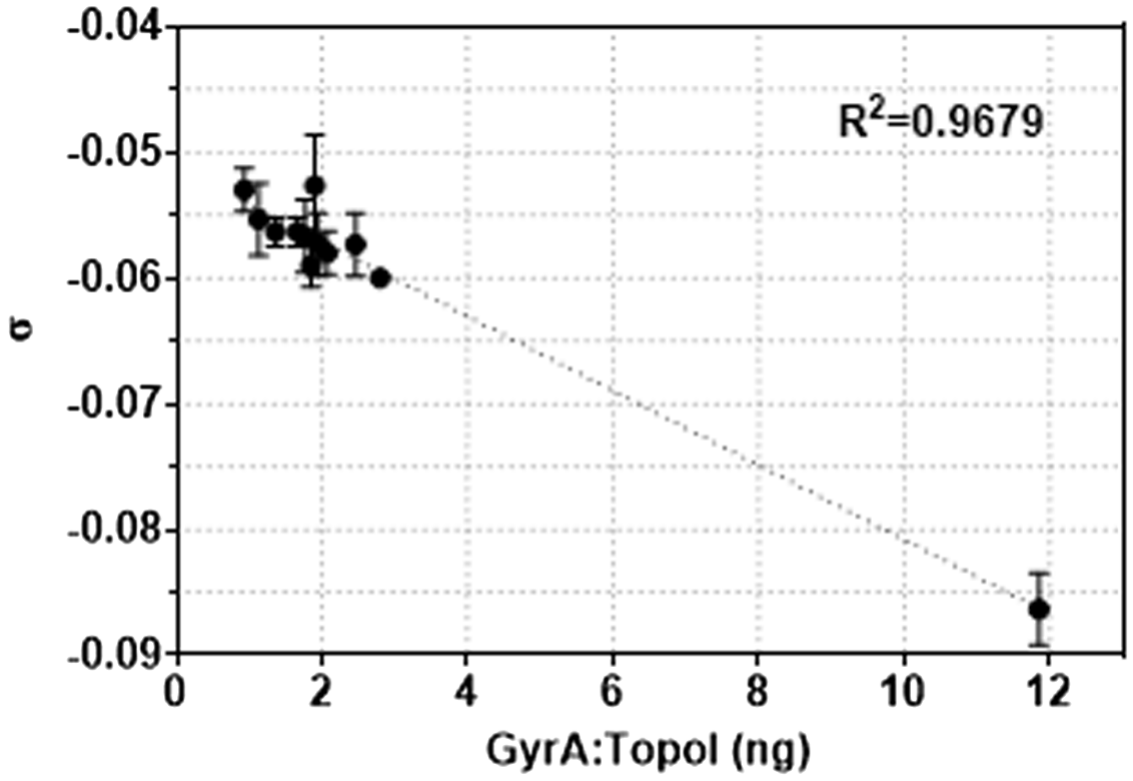
Correlation between GyrA:TopoI ratio and Sc level. Data shown are σ values (mean ± SD of three independent replicates) estimated in 2D-agarose gels and GyrA:TopoI ratio (in ng). Data correspond to samples of strains R6, P_Zn_*topA* and ∆*topA*P_Zn_*topA* grown in different ZnSO_4_ concentrations.

Super-resolution confocal microscopy was used for the first time to estimate compaction of nucleoids. Samples analyzed contained different amounts of TopoI by regulated expression (Figure 5). Three strains representing control situations were studied. Δ*topA*P_Zn_*topA* grown in the absence of ZnSO_4_ showed the highest σ (−0.086) and the lowest TopoI amount (1.5 ng, Table 2). P_Zn_*topA* in the absence of ZnSO_4_ represented the wild-type situation, with an equilibrium σ value of −0.060 and normal TopoI levels (12.2 ng, Table 2). R6 treated with Nov for 30 min represented the most relaxed situation (σ = −0.024). As observed in the confocal micrographs, these strains showed highly compacted (Δ*topA*P_Zn_*topA*), intermediate-compacted (P_Zn_*topA*), and low-compacted (R6 plus Nov) nucleoids (Figure 5A). Quantification of mean gray values of nucleoids in the mentioned strains with different TopoI amounts revealed a good correlation (*R*^2^ = 0.93) between these values and σ values obtained by classic analysis of plasmid topoisomers by 2D-gel electrophoresis (Figures 5B, C). Good correlations were also found between GyrA:TopoI ratio and σ (*R*^2^ = 0.97; Figure 6).

## Discussion

Maintenance of Sc is essential for cell viability, given that Sc regulates all processes in which DNA is involved, including replication and transcription. Two enzymes, gyrase and TopoI, are involved in Sc-level preservation in *S. pneumoniae*. Homeostatic responses to changes in Sc have been observed when Sc density decreases by 25% (Ferrándiz et al., 2010) or when it increases by 40% (Ferrándiz et al., 2016). These responses include transcriptional regulation of topoisomerases. Relaxation triggers upregulation (of about 2-fold) of gyrase genes (*gyrA* and *gyrB*) and downregulation (of about 10-fold) of TopoI (*topA*). However, when Sc increases, *topA*, is also downregulated (about 2-fold), while *gyrA* and *gyrB* remain unchanged. In this way, the downregulation of *topA* transcription, although to different levels, allowed cell growth and the recovery of Sc. However, the possible effects of increasing TopoI levels on Sc and cell viability remained unknown. In this study, we inquired about the effects of *topA* transcription deregulation in Sc and its correlation with cell viability.

Firstly, we estimated the number of topoisomerase molecules per cell in wild type R6 strain under exponential-growth conditions. These figures were of 1,432 for TopoI and 2,184 for GyrA. These molecule numbers allowed an appropriate Sc (σ = −0.057). Given that two molecules of GyrA are present in the gyrase heterodimer GyrA_2_GyrB_2_, these data indicate a number of gyrase enzymes of about 1,092, which represents a ratio of about 1:1.3 for gyrase and TopoI in *S. pneumoniae*. The role of both gyrase and TopoI in transcription (Ahmed et al., 2017; Ferrándiz et al., 2021), and the interaction of TopoI with RNA polymerase (Tiwari et al., 2016; Ahmed et al., 2017; Ferrándiz et al., 2021), is consistent with the equivalent number of TopoI and gyrase molecules per cell. This also supports the twin Sc-domain model, in which negative and positive Sc domains are transiently generated, respectively, behind and ahead of the moving RNA-polymerase (Liu and Wang, 1987). The number of gyrase molecules bound to the chromosome in *E. coli* has been estimated to be 600 (Stracy et al., 2019), lower than our estimation of molecules supporting active growth in *S. pneumoniae*. The size of the pneumococcal chromosome (≈2 Mb) is about half of that of the *E. coli* one. It would be tempting to speculate that the number of chromosome-bound gyrase would be higher in *S. pneumoniae* than in *E. coli*, including those associated with transcriptional machinery. In fact, active transcription constitutes a major architectural feature (Cook and Marenduzzo, 2018) in chromosome organization. Gyrase would play a structural role on chromosome compaction in *S. pneumoniae*, a role normally played by nucleoid-associated proteins in *E.coli*, which are scarce in *S. pneumoniae*.

We have established that the gyrase:TopoI proportion is the main factor contributing to Sc maintenance and viability. In the case of P_Zn_*topA*, since the increase in TopoI induced by ZnSO_4_ was not accompanied by a corresponding increase in gyrase, this GyrA:TopoI proportion varied with the addition of the P_Zn_ inducer to the medium: 1:0.58 for no ZnSO_4_; 1:0.88 for 75 μM; 1:1.05 for 150 μM; and 1:1.26 for 300 μM (Table 2). As there was not a significant change in Sc, this GyrA:TopoI imbalance, especially at 150 and 300 μM ZnSO_4_, must be the cause of the inhibition of growth observed at these ZnSO_4_ concentrations, taking place in a Sc-independent manner.

In the strain Δ*topA*P_Zn_*topA*, in which *topA* has been deleted from its chromosomal location and a copy of *topA* was present under the control of P_Zn_ promoter, the number of TopoI molecules supporting growth (in 75 μM ZnSO_4_) was about 2-fold lower (665) than in the wild type R6 strain. However, the ratio gyrase:TopoI enzymes was maintained near 1:1 due to the decrease in the amount of gyrase in this strain. Furthermore, Sc was similar to that observed in the wild type R6 strain (σ = −0.060 vs. σ = −0.056). The good correlation found between GyrA:TopoI proportion and σ (Figure 6) supports this idea. Nevertheless, a higher proportion, of 1:0.09, was observed in this strain in the absence of TopoI (in the absence of ZnSO_4_) yielding to hyper Sc and lethality. Sc density (−0.080 to −0.086) was >40% higher than that of Δ*topA*P_Zn_*topA* grown in a medium with 150 or 200 μM of ZnSO_4_ (−0.057). However, when this strain was grown in the absence of ZnSO_4_, but in the presence of Nov, a change in σ from −0.080 to −0.068 was observed. This relaxation caused by the inhibition of gyrase by Nov was enough to allow growth, given that the increase in Sc was only 19.3%, lower than 40%, which is the upper limit for viability for *S. pneumoniae* (Ferrándiz et al., 2016). A similar compensatory mechanism targeting gyrase activity has been described in *E. coli*, *S. enterica*, and *S. flexneri*, where some mutations in the gyrase genes that affect the activity of the enzyme suppressed the lethal phenotype of Δ*topA* cells (DiNardo et al., 1982). In the case of *S. pneumoniae*, deletion of *topA* is viable only when it is complemented with an additional copy of the gene under P_Zn_ in those conditions in which it is expressed. We did not find mutations in gyrase genes under no expression of *topA* to compensate the lack of TopoI. However, our CHIP-Seq experiments (Ferrándiz et al., 2016, 2021) showed an *in vivo* interaction of TopoI with the *gyrA* promoter, suggesting a role for TopoI in the transcription of *gyrA*.

In this study, we have been able to measure nucleoid compaction by using super-resolution confocal microscopy. Nucleoids have been previously observed by DAPI staining and phase-contrast microcopy in *S. pneumoniae* (Mercy et al., 2019). We used super-resolution fluorescence microscopy of samples stained with the DNA intercalant Sytox, and considered mean intensity values as a measure of nucleoid compaction. The values estimated in this way for the three strains studied under different growth conditions showed a good correlation with those values of Sc density estimated by 2D-gel electrophoresis of plasmid topoisomers. This result validates the method of 2D-electrophoresis of plasmid topoisomers for the estimation of nucleoid Sc and constitutes the first time that a correlation between Sc density in plasmids and nucleoid compaction has been established. Overall, our results show that an imbalance in the gyrase and TopoI activities leads to nucleoid Sc changes (i.e., compaction) that compromise cell viability. When TopoI is depleted, this equilibrium shifts to an increased negative Sc that is associated with lethality.

We have clearly established that both the gyrase: TopoI ratio and activity are essential to maintain appropriate Sc levels, which is essential for cell viability. The possibility to apply combination therapies with antibiotics targeting topoisomerases, such as fluoroquinolones or seconeolitsine, and RNA polymerase inhibitors, such as rifampicin, remains open.

## Data availability statement

The raw data supporting the conclusions of this article will be made available by the authors, without undue reservation.

## Author contributions

MG-L and M-JF carried out most experiments. DM carried out confocal microscopy. M-JF and AGC conceived, designed, and supervised the study. AGC got funding, administered the project, and wrote the original draft, which was reviewed and edited by M-JF. All authors contributed to the article and approved the submitted version.

## Funding

This work was supported by project PID2021-124738OB-100 to AGC, financed by MCIN/AEI/10.13039/501100011033/FEDER, UE.

## Conflict of interest

The authors declare that the research was conducted in the absence of any commercial or financial relationships that could be construed as a potential conflict of interest.

## Publisher’s note

All claims expressed in this article are solely those of the authors and do not necessarily represent those of their affiliated organizations, or those of the publisher, the editors and the reviewers. Any product that may be evaluated in this article, or claim that may be made by its manufacturer, is not guaranteed or endorsed by the publisher.
